# Strong vacuum squeezing from bichromatically driven Kerrlike cavities: from optomechanics to superconducting circuits

**DOI:** 10.1038/srep21964

**Published:** 2016-02-26

**Authors:** Rafael Garcés, Germán J. de Valcárcel

**Affiliations:** 1 Departament d’Òptica, Facultat de Física, Universitat de València, Dr. Moliner 50, 46100 Burjassot (Valencia), Spain

## Abstract

Squeezed light, displaying less fluctuation than vacuum in some observable, is key in the flourishing field of quantum technologies. Optical or microwave cavities containing a Kerr nonlinearity are known to potentially yield large levels of squeezing, which have been recently observed in optomechanics and nonlinear superconducting circuit platforms. Such Kerr-cavity squeezing however suffers from two fundamental drawbacks. First, optimal squeezing requires working close to turning points of a bistable cycle, which are highly unstable against noise thus rendering optimal squeezing inaccessible. Second, the light field has a macroscopic coherent component corresponding to the pump, making it less versatile than the so-called squeezed vacuum, characterised by a null mean field. Here we prove analytically and numerically that the bichromatic pumping of optomechanical and superconducting circuit cavities removes both limitations. This finding should boost the development of a new generation of robust vacuum squeezers in the microwave and optical domains with current technology.

Quantum fluctuations are perhaps one of the most fascinating consequences of the quantum nature of light. Even in its ground state –the vacuum– the electromagnetic field exhibits fluctuations, which are the analogue of the zero-point fluctuations of a quantum mechanical harmonic oscillator. Being a constitutive part of the field, quantum fluctuations cannot be removed and manifest as nontechnical –quantum– noise in optical experiments. Yet they can be engineered via interactions, as allowed by the pertinent Heisenberg uncertainty relations.

A useful way to characterise quantum fluctuations of light is in terms of the quadratures of the electromagnetic field. Considering a single mode for simplicity, with annihilation and creation operators denoted by *a* and *a*^†^, we define a quadrature^1^ as *q*_*θ*_ = *ae*^−*iθ*^ + *a*^†^*e*^*iθ*^. Experimentally, quadratures are measured via homodyne detection, where the problem light and an intense laser beam of the same frequency (the local oscillator) are combined in a beam-splitter, and the difference between the intensities of the two output ports is measured. The quadrature angle *θ* is selected by varying the phase of the local oscillator. Two orthogonal quadratures form a canonical pair, with commutator [*q*_*θ*_, *q*_*θ*+*π*/2_] = 2*i*, and verify the Heisenberg uncertainty relation Δ*q*_*θ*_ Δ*q*_*θ*+*π*/2_ ≥ 1. For the vacuum Δ*q*_*θ*_ = 1 for all *θ*, which sets the so-called standard quantum limit; laser light, ideally represented by coherent states, is ultimately limited by such (phase-independent) uncertainty level. On the contrary squeezed states of light^1^,^2^ display a phase-dependent quadrature uncertainty, there being a “squeezing angle” *θ* = *θ*_s_ for which 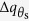 is minimum and less than 1, while 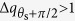.

Squeezed light plays a central role in the fields of quantum information with continuous variables^3^,^4^ and precision measurement^5^,^6^, hence disposing of a variety of squeezing sources is relevant. While optical parametric oscillators, working close to their oscillation threshold, are the best squeezers so far (the present benchmark^7^ is 12.7 dB squeezing, or 95% reduction of vacuum noise), recent experiments with optomechanical (OM) and superconducting circuit (SCC) cavities point to their place in this context^8^,^9^,^10^,^11^,^12^: OM cavities have demonstrated 1.7 dB squeezing^10^ (32% reduction), and SCC cavities have reached 10 dB squeezing (90% reduction)^12^. The generation of strong squeezing in these systems is based on the existence of a bistable cycle^2^,^13^,^14^,^15^,^16^, that appears due to the particular intensity-dependent nonlinearity. At the turning points of bistability a strong reduction of the fluctuations takes place in one quadrature of the light field; however, this squeezing generation presents two handicaps: working close to the turning points makes the system highly unstable against noise^2^,^17^, and the presence of a macroscopic mean field corresponding to the pump makes it less versatile than a squeezed vacuum state^1^,^2^. Unlike OM and SCC cavities, optical parametric oscillators generate a squeezed vacuum whose optimum is reached close to the parametric oscillation threshold, which does not suffer from the discontinuity problems of bistable loops. Such differences come from the different underlying physics in each case: while optical parametric oscillators are based on effective three-photon, or second order, interactions, the quoted experiments in OM and SCC cavities rely on effective four-photon, or third order–Kerr–interactions.

Light squeezing generation is a very active area of research, including single-atom sources^18^, and multi-mode configurations^19^,^20^,^21^. As well squeezing of matter waves has been considered^22^,^23^ and, very especially, of mechanical oscillators in OM systems^24^,^25^,^26^,^27^,^28^,^29^,^30^.

The Kerr effect modifies the refractive index of a medium proportionally to the circulating light intensity (it is a nonlinear optical effect), modifying accordingly the optical thickness of the medium. It is modelled by the Hamiltonian^2^,^13^ 
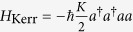
, where *a* is the photon annihilation operator, and *K* is the Kerr coupling constant. When a Kerr medium is placed inside a cavity (be it optical or microwave for our purposes), a shift of the resonances is produced porportionally to the intracavity photon number *a*^†^*a*, in which case *K* is the frequency shift per photon. The Kerr nonlinearity (and, in general, the four-wave mixing), is at the roots of classic squeezing experiments in quantum optics^31^, but its implementation has suffered from parasistic effects (e.g. spontaneous emission in atomic gases) that degrade the quality of squeezing. Clean implementations of the Kerr effect are observed in SCC containing Josephson junctions (due to their nonlinear inductance)^12^,^32^,^33^, where *H*_Kerr_ is the simplest interaction Hamiltonian. In OM systems, where electromagnetic and mechanical degrees of freedom interact, the Kerr interaction is effective as follows. The standard OM cavity model, which successfully accounts for most of the experiments to date^34^, involves a single cavity mode and a single mechanical oscillator (of frequency *ω*_m_) interacting via radiation pressure. The corresponding interaction Hamiltonian reads^34^ *H*_OM_ = −*ħg*_0_*xa*^†^*a*, where *x* is the displacement of the mechanical oscillator from its equilibrium, measured in units of its zero-point fluctuation amplitude (*x*_zpf_), and *g*_0_ is the cavity resonance shift for a mechanical amplitude of *x*_zpf_ (so-called vacuum OM coupling strength^34^)(Supplementary Information). Kerr and OM cavities are analogous^15^,^16^,^35^ because the radiation pressure force *F*_RP_ ≡ −∂*H*_OM_/∂*x* = *ħg*_0_*a*^†^*a* displaces the movable mechanical element proportionally to the intracavity photon number *a*^†^*a*, similarly to the optical cavity length variation occuring in cavities containing optical Kerr media. *H*_OM_ describes a large variety of OM cavities, including suspended micromirrors and membranes, microtoroids, defected photonic crystals, nanorods, etc^34^.

The rationale behind this work is threefold. First, OM and SCC cavities can behave as Kerr cavities. Second, there is a recent prediction that Kerr cavities driven by two close frequencies^36^,^37^ do not show the classic bistable response of monochromatic driving^15^,^16^,^17^,^34^,^38^,^39^, but instead undergo a degenerate four-wave mixing bifurcation where the (non-injected) mean frequency starts oscillating spontaneously, similarly to the optical parametric oscillator threshold. Finally there is a well-known relationship between bifurcations and squeezing in optical cavities^13^. Therefore in this work we consider both OM and SCC Kerrlike cavities driven by bichromatic fields.

## Results and Discussion

### Model

When the two driving frequencies, which we denote by *ω*_L_ ± Ω, are sufficiently close to the same cavity resonance frequency *ω*_cav_, a single cavity mode is excited. Hence the corresponding SCC Kerr-cavity model coincides with the standard one^12^,^14^,^32^,^33^, the only difference being in the form of the driving. As well, as shown in the Methods Section, the OM model reduces to a Kerrlike model when the mechanical resonance frequency *ω*_m_ is simultaneously much larger than the cavity photon damping rate which we denote by 2*κ* (resolved-sideband limit), and the modulation frequency Ω, in which case the mechanical dynamics can be adiabatically eliminated. Considering the injection of two equally intense coherent lines, of normalised amplitudes 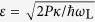 (*P* is the total power coupled to the cavity), the Heisenberg-Langevin equation for the field, in a rotating frame at frequency *ω*_L_, reads


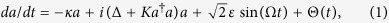


where Δ = (*ω*_L_ − *ω*_cav_) is the detuning between the (non-injected) mid-frequency of the driving and the cavity frequency, *K* is the Kerr coefficient of the SCC cavity^32^ or 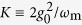 is an effective Kerr coupling constant in the OM case, and Θ(*t*) is a noise term: in the SCC case 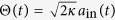 with *a*_in_(*t*) a white Gaussian quantum noise coming from vacuum fluctuations entering the cavity; in the OM case 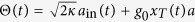, being *x*_*T*_ the mechanical displacement fluctuation caused by zero-point and thermal agitation (Methods Section). Equation (1) provides a unified description of SCC and OM cavities. It can be considered as the model equation for a Josephson amplifier^14^,^32^ or as an approximation for the OM cavity under the conditions set above, whose validity will be assessed later. Modulated OM cavities have been considered in the literature in order to obtain interesting mechanical effects^40^,^41^,^42^,^43^, which occur when the modulation frequency Ω is a multiple of the mechanical frequency *ω*_m_. However we anticipate that such “harmonic” driving does not lead to light squeezing.

Next we analyse the dynamics of (1) in the semiclassical and linear approximations as usual: we split the operators into a mean field part, 〈*a*〉 = *α*(*t*), plus a fluctuation, i.e. *a* = *α*(*t*) + *δa*, and disregard nonlinear terms in the fluctuations.

### Mean field solution

**Figure 1:**
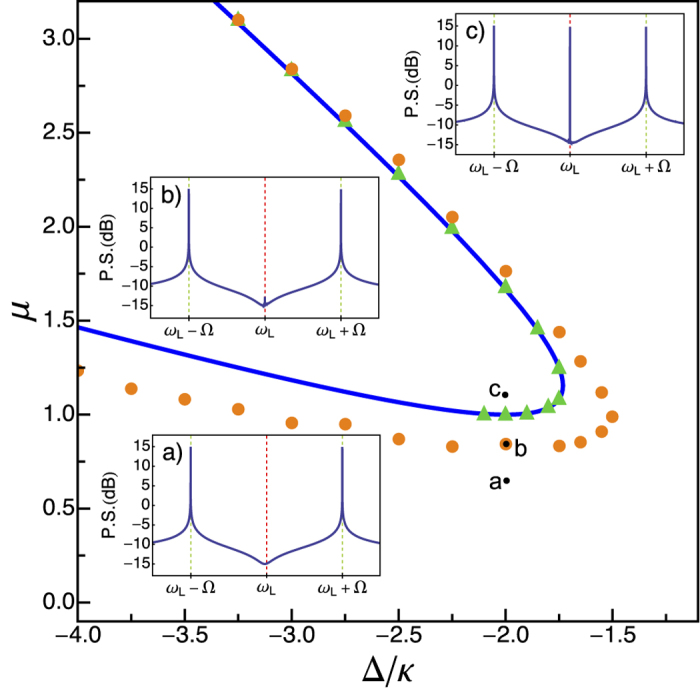
Semiclassical bifurcation diagram of the bichromatically driven optomechanical and superconducting circuit cavities. A positive Kerr coupling constant *K* has been used; for *K* < 0 the result is identical, upon swapping Δ → −Δ. *μ* is proportional to the injection power and Δ/*κ* is the ratio of the cavity detuning to the photon damping rate. The base solution becomes unstable inside the tongue (3), where the noninjected frequency *ω*_L_ appears. The full, blue line represents the analytical prediction based on model (1). Symbols denote boundaries obtained from numerical integration of the mean field equations of the Kerr model (green diamonds), which actually represent a superconducting circuit cavity, and of the complete optomechanical model (orange circles). The insets show the optical power spectrum (logarithmic scale) for different injection parameters: (**a**) below the lower signal oscillation threshold (base solution), (**b**) a small signal at *ω*_L_ emerges close above the lower signal oscillation threshold and (**c**) the signal is fully developed well inside the tongue. The red line denotes the location of the carrier frequency *ω*_L_ and the two main peaks located at *ω*_L_ ± Ω correspond to the driving. The modulation frequency Ω/*κ* = 4*π*. In the optomechanical case the actual parameters are *ω*_m_/*κ* = 30, *Q*_m_ = 10^5^.

The mean field equation reads 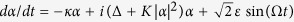, which has been studied in the context of optical pattern formation^36^,^37^. This equation admits periodic solutions of the form 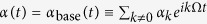, which we call base solutions as they exist always. Note that they do not contain a constant term (*k* ≠ 0), meaning that there is no mean field at the optical frequency *ω*_L_; hence the type of squeezing we describe next around *ω*_L_ is squeezed vacuum. *α*_base_(*t*) can be computed numerically and can be a complicated function of time in general. In order to gain analytical insight we consider the limit Ω ≫ *κ*,|Δ|, in which case 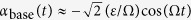 (Supplementary Information). The intracavity mean photon number in the base state is


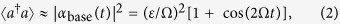


which shows a linear dependence with the injection power *ε*^2^, and hence no bistability. The base solution needs not be stable for all parameter settings, as a standard linear stability analysis reveals (Methods Section and Supplementary Information): for 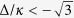 (red detuning side), *α*_base_(*t*) becomes unstable between a lower and an upper injection power. Expressed in terms of a normalised injection parameter *μ* ≡ *Kε*^2^/*κ*Ω^2^, *α*_base_(*t*) is unstable for *μ*^↓^ < *μ* < *μ*^↑^, where


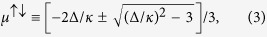


defines a “tongue” on the plane *μ* − Δ/*κ* (Fig. 1). Just at *μ* = *μ*^↓^ or *μ* = *μ*^↑^, a bifurcation gives rise to the emergence of a constant, bias component on top of *α*_base_(*t*). Such component corresponds to an emission line at *ω*_L_, which comes from the degenerate four-wave mixing process (*ω*_L_ + Ω) + (*ω*_L_ − Ω) → *ω*_L_ + *ω*_L_. As a consequence the new component at *ω*_L_ is phase locked to the base solution, displaying phase bistability between two opposite phase values^36^,^37^, as it happens in the degenerate optical parametric oscillator above threshold. We have verified this prediction by numerical integration of the Kerrlike model (1) as well as of the complete OM model without approximations, under different parametric conditions, finding very good quantitative agreement (Fig. 1).

### Spectrum of squeezing

Our actual goal is to demonstrate strong vacuum squeezing at the degenerate four-wave mixing bifurcation, in particular when it is approached from outside the instability tongue. As there the mean field at frequency *ω*_L_ is null, the squeezing we describe next corresponds to a squeezed vacuum. The quantity of interest is the so-called squeezing spectrum^1^,^2^ *S*_*θ*_(*ω*), which measures the noise power spectral density (spectral variance) of the light quadrature *q*_*θ*_ ≡ *e*^−*iθ*^*a* + *e*^*iθ*^*a*^†^ leaving the cavity, at a noise frequency *ω*. It is normalised so that in the vacuum state *S* = 1, while if *S*(*ω*_*s*_) < 1 there is squeezing at the noise frequency *ω*_*s*_ (*S* = 0 marks perfect squeezing: no fluctuation at all). The spectrum *S*_*θ*_(*ω*) is measured experimentally by homodyning^1^,^2^,^8^,^9^,^10^,^12^ the light leaving the cavity with a strong local oscillator signal of frequency *ω*_L_.

#### Analytical prediction

As shown in the Supplementary Information, 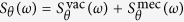, where 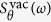 comes from the field vacuum fluctuations, while 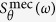 comes from mechanical fluctuations and is present only in the OM case. Just at the bifurcation (*μ* = *μ*^↑^ or *μ* = *μ*^↓^), the strongest squeezing is observed as expected. Its optimum value, obtained by adjusting the quadrature angle *θ*, follows from


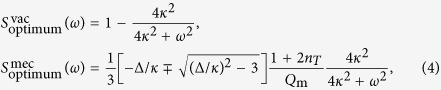


where (remind: 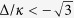), the subscript 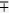 corresponds to *μ* = *μ*^↑↓^, respectively, *n*_*T*_ ≡ [exp(*ħω*_m_/*k*_B_*T*) − 1]^−1^ is the mean number of thermal phonons at temperature *T*, with *k*_B_ the Boltzmann constant, and *Q*_m_ is the mechanical resonance quality factor. *S*^vac^ has been computed ignoring thermal photons. This is an excellent approximation in the optical domain, and in the microwave domain at cryogenic temperatures. If thermal photons are considered the expression for *S*^vac^ reads as in (4), but multiplied by (1 + 2*n*_*L*_), where *n*_*L*_ ≡ [exp(*ħω*_L_/*k*_B_*T*) − 1]^−1^ is the mean number of thermal photons.

Perfect squeezing is ideally predicted in the SCC case at zero noise frequency, *S*_optimum_(0) = 0, with a bandwidth equal to 2*κ*. In the OM case *S*_optimum_(0) > 0 because 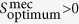. Nevertheless *S*_optimum_(0) can be much less than 1 in the OM case because of the large values of *Q*_m_ ~ 10^5^ − 10^6^ attained in experiments^34^. Large *n*_*T*_ values are obviously deleterious, however they can be made very small (*n*_*T*_ ≪ 1) via the so-called sideband cooling^34^,^44^,^45^,^46^, which has been used to improve optical squeezing in recent OM experiments^10^.

It is interesting to note that our result for the optimum squeezing attainable (4) coincides with the one that can be obtained in the usual Kerr-like systems with monochromatic driving, see the Methods Section. However the physical situation is very different in both cases: while in the bichromatic case analysed here *S*_optimum_ is reached at a continuous bifurcation point, in the monochromatic case it requires to work at the turning points of the bistable cycle, with all the associated problems discussed above. Another important difference is that in the monochromatic case the squeezing is produced at the injection frequency, where there is a strong mean field present.

#### Numerical results

**Figure 2:**
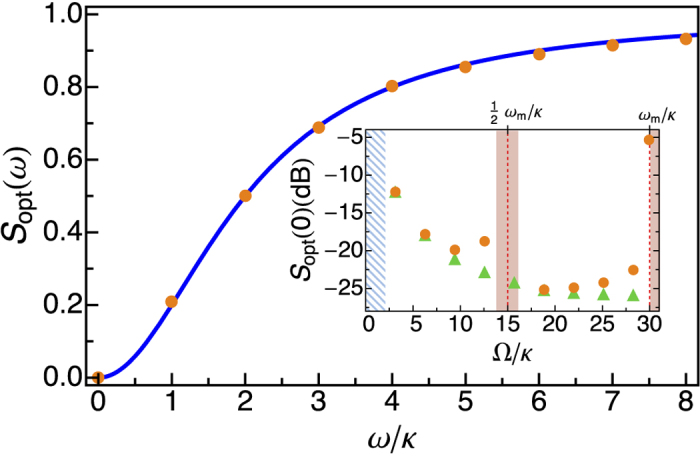
Squeezing spectrum. The full, blue line represents the best squeezing spectrum of the OM cavity, according to the analytical prediction from model (1), for Ω/*κ* = 4*π*, Δ/*κ* = −2, and *n*_*T*_ = 0. Orange symbols denote the results of numerical simulations of the complete optomechanical problem for the same parameters as in Fig. 1, with Δ/*κ* = −2 (close below point (b) in Fig. 1; *μ* = 0.837). The inset shows the dependence of the numerically obtained optimal squeezing on the modulation frequency Ω, of both superconducting circuit cavities (green diamonds) and optomechanical cavities (orange circles). Parameters as in Fig. 1. Within the left shadowed region the mechanism proposed here does not work (a minimum modulation frequency, around Ω/*κ* = 2*π*, is needed), while in the middle and right ones it does not only for the optomechanical cavity: the rightmost orange circle corresponds to Ω = 29.9*κ*, slightly less than the mechanical resonance frequency *ω*_m_ = 30*κ*; for Ω = *ω*_m_ the effect is completely lost, while for Ω > *ω*_m_ the scenario completely changes and a different description is needed.

In order to check that the different approximations used do not lead to artificially low levels of noise reduction, we have computed *S*_*θ*_(*ω*) numerically from the full OM and the Kerr model equations using realistic parameter values (Supplementary Information), finding excellent agreement as shown in Fig. 2. In the SCC cavity case the results point to a monotonic improvement of the squeezing as Ω is increased. In the OM case the mechanical resonance plays a clear role: the effect is lost for modulation frequencies Ω close to *ω*_m_/2, and is clearly degraded as Ω approaches *ω*_m_. These phenomena have their roots in the fact that the driving force acting on the mechanical element is the photon number given in (2). Thus, for Ω = *ω*_m_/2 a resonant forcing of the mechanical oscillator occurs (1:1 resonance) and for Ω = *ω*_m_ the mechanical element is driven at twice its frequency (2:1 resonance), known as parametric resonance. So, high levels of squeezing of light are predicted for Ω < *ω*_m_, excluding the regions around Ω = *ω*_m_/2 and Ω = *ω*_m_ where the effect is lost; while for Ω > *ω*_m_ the scenario completely changes and a different theoretical description is needed.

## Conclusions

In summary, we have shown that a bichromatic driving of a Kerr-like system, such as an optomechanical cavity or a nonlinear superconducting circuit, can produce a strong reduction of the fluctuations for one quadrature of the electromagnetic field.

The bichromatic driving produces a change of the bistable behaviour that happens with a monochromatic driving to a kind of degenerate four-wave mixing process, where the injected signals at frequencies *ω*_L_ + Ω and *ω*_L_ − Ω give rise to a component at the non-injected frequency *ω*_L_, (*ω*_L_ + Ω) + (*ω*_L_ − Ω) → *ω*_L_ + *ω*_L_, at the bifurcation given by (3), see Fig. 1.

When approaching the bifurcation from outside the “tongue” (Fig. 1), we have shown that there is a strong optical quadrature squeezing when homodying the output field with a local oscillator at frequency *ω*_L_. As, outside the “tongue”, there is no mean field at that frequency, the predicted squeezing corresponds to a vacuum squeezing. The system has independent noise terms coming from electromagnetic vacuum field fluctuations and from mechanical thermal fluctuations in the OM case, so the spectrum of squeezing can be decomposed as 
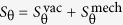
 (eq. (4)). When the best squeezing is reached, at the bifurcation and for *ω* = 0, the vacuum part becomes null *S*_optimum_(0) = 0 and the mechanical part in the OM case is very small even for a moderate number of thermal phonons, due to the high mechanical quality factors (*Q*_m_) that can be attained in the experiments.

## Methods

### OM Kerrlike model

For the optomechanical case we begin with the usual Langevin equations, but with a bicrhomatic driving


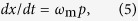



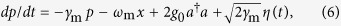



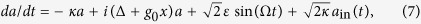


where the overdot indicates time derivative. Here *a*_in_(*t*) and *η*(*t*) are white Gaussian noises of zero mean, whose only non-null two-time correlations read


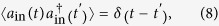



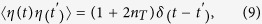


where *n*_*T*_ = [exp(*ħω*_m_/*k*_B_*T*) − 1]^−1^ is the mean number of thermal phonons at temperature *T*, with *k*_B_ the Boltzmann constant. This form for the mechanical noise correlator is valid in the mechanical high-Q limit (*Q*_m_ = *ω*_m_/*γ*_m_ ≫ 1), which we assume.

The equations for the mechanical element (5, 6) can be formally solved in the Fourier domain, obtaining that


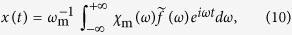


where 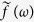 is the Fourier transform of the driving force 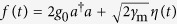 and 
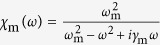
 is the mechanical susceptibility. In the limit *ω*_m_ ≫ *κ*, Ω the Fourier transform of the radiation pressure force term 2*g*_0_*a*^†^*a*, contains only low frequencies as compared to *ω*_m_, since the cavity acts as a low-pass filter of width 2*κ*, so for this term we can make the approximation *χ*_m_(*ω*) → *χ*_m_(0) = 1 in equation (10).

Thus, the displacement can be written as *x* = 2*g*_0_*a*^†^*a*/*ω*_m_ + *x*_*T*_ where *x*_*T*_ is a fluctuation due to the mechanical noise with autocorrelation 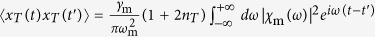.

After substitution of *x* in the field equation (7), the optomechanical Kerr-like approximation (eq. (1)) for our model is obtained. For more details see the Supplementary Information.

### Fluctuation dynamics

The equations for the fluctuations *δa* = *a* − *α*_base_(*t*) and 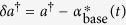 are obtained trivially from equation (1) and by neglecting the nonlinear fluctuating terms, as well as the fast oscillating terms at 2Ω, in a kind of a rotating wave approximation. As discussed in the Supplementary Information the dynamical fluctuation equations can be cast in matrix form as:


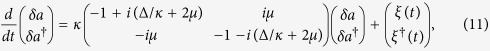


with 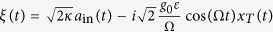 a noise term, where the first part is common in both problems (SCC and OM systems) and the second term only appear in the OM case. Note that equation (11) corresponds to a parametric process in which *μ* plays the role of a parametric gain, and also of an extra detuning.

The stability analysis of the base solution follows by analysing the eigenvalues of the coefficient matrix in (11), which read 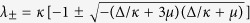. When at least one of these eigenvalues becomes positive the base solution becomes unstable. The region of instability is given by the *μ*^↑↓^ parameter, computed making *λ*_+_ = 0.

#### The monochromatic driving case

The usual OM and SCC Kerr-like with monochromatic driving are described by a similar equation to (1), but changing the driving part 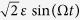, giving


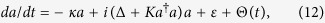


with the same definitions as in the Model Section.

Its corresponding mean field equation reads *dα*/*dt* = −*κα* + *i*(Δ + *K*|*α*|^2^)*α* + *ε*, whose steady state solutions *α*_s_ can be easily computed making *dα*/*dt* = 0. Thus, the intracavity mean photon number is identified as 〈*a*^†^*a*〉 = |*α*_s_|^2^ = *I*_s_.

The system presents a bistable behaviour for 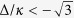. There is an interval of the driving intensity where there are two stable intracavity mean photon number solutions for the same value of the driving intensity. These two stable branches of solutions are connected by an unstable branch. The system destabilizes at the turning points of the bistable cycle given by


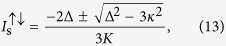


and it is in these points where the best squeezing is obtained.

The fluctuation dynamics is given too by equation (11) but with the following changes: i) *μ* = (*K*/*κ*)*I*_s_ and ii) the noise term being 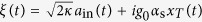.

### Computation of the spectrum of squeezing

To compute the spectrum of squeezing we need to solve the system of equations for the fluctuation dynamics. We note that the left eigenvectors of the coefficient matrix in (11) can be written, near the bifurcation, as 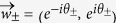, where *θ*_+_ = −*θ*_−_. Thus projecting equation (11) onto 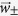 from the left yields decoupled equations for the intracavity quadrature fluctuations 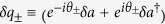,


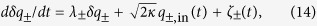


where 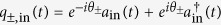 is the corresponding quadrature vacuum noise and


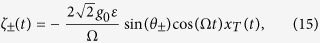


is the mechanical noise coupled to that quadrature. Note that either *θ*_±_ should be zero (amplitude quadrature) mechanical noise would have no effect on that quadrature, as is well known for radiation pressure driven optomechanics.

We are interested in the spectral variance, called squeezing spectrum, of the outgoing detected quadrature, FORMULA, which is calculated from the two-time correlations 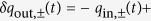

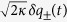
, 


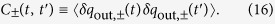


After straightforward algebra can be written as


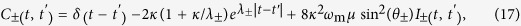


where the last term is only valid for the OM case, with


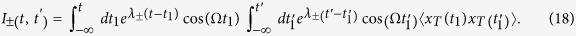


From equation (17) it can be seen that in the OM case the correlator is not stationary, due to the last term corresponding to the mechanical noise. Thus, the spectrum of squeezing has to be computed using the following definition^47^


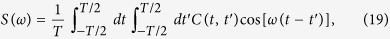


where *T* is the measurement time. As shown in the Supplementary Information *S*(*ω*) can be worked out analytically.

## Additional Information

**How to cite this article**: Garcés, R. and de Valcárcel, G. J. Strong vacuum squeezing from bichromatically driven Kerrlike cavities: from optomechanics to superconducting circuits. *Sci. Rep.* **6**, 21964; doi: 10.1038/srep21964 (2016).

## Supplementary Material

Supplementary Information
