# Supplementary material for: Strong vacuum squeezing from bichromatically driven Kerrlike cavities: from optomechanics to superconducting circuits
Source: Sci Rep. 2016 Feb 26;6:21964. doi: 10.1038/srep21964 (PMC4768168; doi:10.1038/srep21964)
Supplement: Supplementary Information [file srep21964-s1.pdf]

Supplementary Information

Strong vacuum squeezing from bichromatically driven Kerrlike cavities: from optomechanics to superconducting circuits

Rafael Garcés[1*] and Germán J. de Valcárcel[1]

[1]*Departament d'Òptica, Facultat de Física,*

Universitat de València, Dr. Moliner 50,

46100 Burjassot (Valencia), Spain,

[*]*rafael.garces@uv.es*

I. THE OPTOMECHANICAL CAVITY MODEL

We consider the standard optomechanical (OM) cavity model[1–3] in which an electromagnetic cavity mode interacts with a mechanical oscillator via radiation pressure. We denote by x and p the mechanical displacement and momentum, respectively, divided by their zero-point fluctuations, $x_{\mathrm{ZPF}} = \sqrt{\hbar/2m_{\mathrm{eff}}\omega_{\mathrm{m}}}$ and $p_{\mathrm{ZPF}} = \hbar/2x_{\mathrm{ZPF}}$ ($\hbar$ is Planck constant divided by 2π, m_{eff} is the effective mass of the mechanical resonator and $\omega_{\mathrm{m}}/2\pi$ is its frequency), and by a and $a^{\dagger}$ the photon annihilation and creation operators of the cavity mode, respectively. The cavity mode is driven by a bichromatic (say optical) field of frequencies $\omega_{\mathrm{L}} \pm \Omega$. Working in the field interaction picture where the electric field operator is proportional to $i\left(ae^{-i\omega_{\mathrm{L}}t} - a^{\dagger}e^{i\omega_{\mathrm{L}}t}\right)$, the total Hamiltonian for the system can be written as

$$H = -\hbar\Delta a^{\dagger}a + \frac{1}{4}\hbar\omega_{\mathrm{m}}\left(x^2 + p^2\right) + H_{\mathrm{OM}} + H_{\mathrm{drive}}, \tag{1}$$

where $\Delta = (\omega_{\mathrm{L}} - \omega_{\mathrm{cav}})$ is the injection-cavity detuning, $H_{\mathrm{OM}} = -\hbar g_0 x a^{\dagger}a$ is the OM interaction Hamiltonian, with g_0 the vacuum optomechanical coupling strength ($g_0 = g x_{\mathrm{ZPF}}$, with g the cavity resonance frequency shift per unit mechanical displacement), and $H_{\mathrm{drive}} = i\hbar\sqrt{2}\epsilon\sin(\Omega t)\left(a^{\dagger} - a\right)$ describes the bichromatic driving. Both the cavity mode and the mechanical oscillator are affected by damping, whose rates we denote respectively by κ (2κ represents the photon loss rate from the cavity, or cavity linewidth) and γ_{m}. These dampings couple the OM cavity to its environment, which manifests as (quantum) noise. Note that we consider the injection of two equally intense lines for simplicity, of amplitudes $\epsilon/\sqrt{2} = \sqrt{\kappa P/\hbar\omega_{\mathrm{L}}}$, where P is the total power (actually coupled to the cavity), and we take ϵ real without loss of generality (this sets a reference phase).

The Heisenberg-Langevin equations of the OM system cavity be written as[1–5]

$$\dot{x} = \omega_{\mathrm{m}} p, \tag{2a}$$

$$\dot{p} = -\gamma_{\mathrm{m}} p - \omega_{\mathrm{m}} x + 2g_0 a^{\dagger}a + \sqrt{2\gamma_{\mathrm{m}}}\eta(t), \tag{2b}$$

$$\dot{a} = -\kappa a + i(\Delta + g_0 x)a + \sqrt{2}\epsilon\sin(\Omega t) + \sqrt{2\kappa}a_{\mathrm{in}}(t), \tag{2c}$$

where the overdot indicates time derivative. Here $a_{\mathrm{in}}(t)$ and $\eta(t)$ are white Gaussian noises of zero mean, whose only non-null two-time correlations read

$$\left\langle a_{\mathrm{in}}(t)\, a_{\mathrm{in}}^{\dagger}(t')\right\rangle = \delta(t - t'), \tag{3a}$$

$$\langle \eta(t)\, \eta(t')\rangle = (1 + 2n_T)\,\delta(t - t'), \tag{3b}$$

where $n_T = \left[\exp\left(\hbar\omega_{\rm m}/k_{\rm B}T\right) - 1\right]^{-1}$ is the mean number of thermal phonons at temperature T, with $k_{\rm B}$ the Boltzmann constant. This form for the mechanical noise correlator is valid in the mechanical high-Q limit ($Q_{\rm m} = \omega_{\rm m}/\gamma_{\rm m} \gg 1$), which we assume.

A. The mechanical susceptibility

The mechanical equations (2a), (2b) can be combined into the usual equation for a damped and forced harmonic oscillator

$$\ddot{x} + \gamma_{\rm m}\dot{x} + \omega_{\rm m}^2 x = \omega_{\rm m} f\left(t\right), \tag{4}$$

where $f = 2g_0 a^\dagger a + \sqrt{2\gamma_{\rm m}}\eta\left(t\right)$. Note that, whichever the form of the force f be, equation (4) admits the formal solution

$$x\left(t\right) = \omega_{\rm m}^{-1}\int_{-\infty}^{+\infty}\chi_{\rm m}\left(\omega\right)\tilde{f}\left(\omega\right)e^{i\omega t}d\omega, \tag{5}$$

where

$$\chi_{\rm m}\left(\omega\right) = \frac{\omega_{\rm m}^2}{\omega_{\rm m}^2 - \omega^2 + i\gamma_{\rm m}\omega}, \tag{6}$$

is the mechanical susceptibility, and $\tilde{f}\left(\omega\right) = \left(2\pi\right)^{-1}\int_{-\infty}^{+\infty} f\left(t\right)e^{-i\omega t}dt$ is the force Fourier transform. In the following it will prove useful splitting the displacement as

$$x = x^{\rm RP} + x_T\left(t\right), \tag{7}$$

where $x^{\rm RP}$ is due to the radiation pressure force only ($f = 2ga^\dagger a$), and $x_T\left(t\right)$ is a fluctuation due to the mechanical noise.

B. Displacement fluctuation due to mechanical noise

The fluctuation $x_T\left(t\right)$ can be calculated from expression (5), using $f\left(t\right) = \sqrt{2\gamma_{\rm m}}\eta\left(t\right)$. It is a coloured Gaussian noise of zero mean and autocorrelation,

$$\left\langle x_T\left(t\right)x_T\left(t'\right)\right\rangle = \frac{\gamma_{\rm m}}{\pi\omega_{\rm m}^2}\left(1 + 2n_T\right)\int_{-\infty}^{+\infty} d\omega\left|\chi_{\rm m}\left(\omega\right)\right|^2 e^{i\omega\left(t - t'\right)}, \tag{8}$$

as follows from $\left\langle\tilde{\eta}\left(\omega\right)\tilde{\eta}\left(\omega'\right)\right\rangle = \left(1 + 2n_T\right)\left(2\pi\right)^{-1}\delta\left(\omega + \omega'\right)$, which derives from the correlation (3b).

II. THE KERRLIKE MODEL

We analyse here the OM model in the limit $\omega_{\mathrm{m}} \gg \kappa, \Omega$, in which the displacement x^{RP} can be adiabatically eliminated as we discuss next, and a single equation for the field, with an effective Kerr nonlinearity, is obtained. The displacement is given by equation (4), with $f = 2g_0 a^\dagger a$. The point is that the photon number $N \equiv a^\dagger a$ contains frequencies which are small as compared to the mechanical frequency ω_{m} in the considered limit. On one hand the photon number expectation value $\langle N \rangle = |\langle a \rangle|^2$ oscillates at frequencies which are multiples of 2Ω, equation (2c), and high harmonics (which could eventually approach ω_{m}) are strongly attenuated because the cavity acts as a low-pass filter of width 2κ. On the other hand the fluctuation δN, which is equal to $\langle a \rangle \delta a^\dagger + \langle a^\dagger \rangle \delta a$ in the linear approximation, also is a low frequency quantity because the field fluctuation δa is again filtered by the cavity. Consequently the Fourier transform $\tilde{N}(\omega)$ of the photon number only has low frequencies as compared to ω_{m}, and we can make the approximation $\chi_{\mathrm{m}}(\omega) \to \chi_{\mathrm{m}}(0) = 1$ in equation (5), getting the result

$$x^{\mathrm{RP}} = \frac{2g_0}{\omega_{\mathrm{m}}} a^\dagger a, \tag{9}$$

which represents an adiabatic elimination of x^{RP}. In such case the model reduces to equation (2c) with x replaced by (7), and x^{RP} given by (9), i.e.

$$\dot{a} = \left[-\kappa + i\left(\Delta + g_0 x_T\right)\right] a + iK a^\dagger a^2 + \sqrt{2}\epsilon \sin(\Omega t) + \sqrt{2\kappa} a_{\mathrm{in}}(t), \tag{10}$$

where we defined $K \equiv \frac{2g_0^2}{\omega_{\mathrm{m}}}$. This is the Kerrlike model analysed in the main text.

There is only one exception to the validity of the above adiabatic elimination of x. This occurs when the so-called parametric instability (a Hopf bifurcation)[1–4,6–9] appears in the system, in which case fluctuations have frequencies on the order of ω_{m}. Such instability however requires strong enough injection to occur and we have checked that does not affect the phenomena we treat here, as we illustrate below.

A. The base solutions

Here we are interested in determining the mean field solutions to equation (10). Expressing the field operators as an expectation value plus a fluctuation, and ignoring nonlinear terms in the fluctuations (linear approximation), the following equation for the mean field

$\langle a \rangle \equiv \alpha(t)$ is obtained:

$$\dot{\alpha} = \left[-\kappa + i\left(\Delta + K|\alpha|^2\right)\right]\alpha + \sqrt{2}\epsilon \sin(\Omega t). \tag{11}$$

This equation admits periodic solutions of the form $\alpha(t) = \alpha_{\text{base}}(t) \equiv \sum_{k\neq 0} \alpha_k e^{ik\Omega t}$, which we call base solutions as they exist always. Note that they are characterised by an absence of a constant term ($k \neq 0$), which physically means that the base solutions do not contain the (noninjected) frequency ω_{L}. Note that this is not peculiar of the Kerr limit, but is a general property of the OM model (2a)-(2c).

The base solutions can be computed numerically and can be complicated functions of time. However there is at least a limit where simple analytical approximations for $\alpha_{\text{base}}(t)$ can be derived. In particular, if $\Omega \gg \kappa, |\Delta|, K|\alpha|^2$, then $\dot{\alpha} \approx \sqrt{2}\epsilon \sin(\Omega t)$, and $\alpha_{\text{base}}(t) \approx -\sqrt{2}(\epsilon/\Omega)\cos(\Omega t)$. Clearly one can use in principle a driving frequency Ω much larger than κ and $|\Delta|$, but what about $K|\alpha_{\text{base}}|^2$? As demonstrated in the next subsection, $K|\alpha_{\text{base}}|^2$ is on the order of κ in the region of interest, hence the initial assumption proves correct a posteriori.

In any case, note that one can choose $\alpha_{\text{base}}(t) = -\sqrt{2}(\epsilon/\Omega)\cos(\Omega t)$ as an exact solution, just by replacing the forcing term $\sqrt{2}\epsilon \sin(\Omega t)$ in equation (11) by a term

$$E(t) = \sqrt{2}\epsilon\left[\sin(\Omega t) + \frac{-\kappa + i\Delta + i3KR^2}{\Omega}\cos(\Omega t) + i\frac{KR^2}{\Omega}\cos(3\Omega t)\right], \tag{12}$$

where $R = \frac{1}{2}\left(\frac{\epsilon}{\Omega}\right)^2$. Note that $E(t)$, as well as the considered driving, is characterised by an absence of a constant term, which would correspond to an injection at the frequency ω_{L}.

B. Linear stability analysis of the base solution

The linear stability of the base solution is assessed by studying the dynamics of the field fluctuations $\delta a = a - \alpha_{\text{base}}(t)$ and $\delta a^\dagger = a^\dagger - \alpha^*_{\text{base}}(t)$. From the field equation (10) we obtain the linearised equation

$$\begin{aligned}\delta\dot{a} &= -\kappa\delta a + i(\Delta + 2\kappa\mu)\delta a + i\kappa\mu\delta a^\dagger + i\kappa\mu\cos(2\Omega t)\left(2\delta a + \delta a^\dagger\right) \\ &+ \sqrt{2\kappa}a_{\text{in}}(t) - i\sqrt{2}\frac{g_0\epsilon}{\Omega}\cos(\Omega t)\,x_T(t),\end{aligned} \tag{13}$$

where we defined the dimensionless injection parameter

$$\mu \equiv \frac{K\epsilon^2}{\kappa\Omega^2} = \frac{2g_0^2\epsilon^2}{\kappa\omega_{\text{m}}\Omega^2}. \tag{14}$$

As μ turns out to be of order 1 in the region of interest (proven below) and we are assuming $\Omega \gg \kappa, |\Delta|$, the term containing $\cos(2\Omega t)$ is highly oscillating and can be neglected in a kind of rotating-wave approximation. Ignoring that term, equation (13), plus the one for $\delta a^\dagger$, can be cast in matrix form as

$$\frac{d}{dt}\begin{pmatrix}\delta a\\ \delta a^\dagger\end{pmatrix} = \kappa\begin{pmatrix}-1+i(\Delta/\kappa+2\mu) & i\mu\\ -i\mu & -1-i(\Delta/\kappa+2\mu)\end{pmatrix}\begin{pmatrix}\delta a\\ \delta a^\dagger\end{pmatrix} + \begin{pmatrix}\xi(t)\\ \xi^\dagger(t)\end{pmatrix}, \tag{15}$$

with $\xi(t) = \sqrt{2\kappa}a_{\text{in}}(t) - i\sqrt{2}\frac{g_0\epsilon}{\Omega}\cos(\Omega t)\, x_T(t)$ a noise term.

Here we are just interested in knowing whether fluctuations are damped, and then the actual state of the system consists of the base solution plus fluctuations, or fluctuations are amplified, in which case they grow without bound (in the linear approximation). In the latter case what the analysis is telling us is that the base solution is no more a stable solution of the system, and is replaced by a new mean field solution. In such case equation (15) would cease to be correct for calculating the evolution of fluctuations and one should derive new equations for the fluctuations, now around the new state, if wanted. The stability problem is solely determined by the eigenvalues of the matrix in equation (15), which read

$$\lambda_\pm = \kappa\left[-1 \pm \sqrt{-(\Delta/\kappa+3\mu)(\Delta/\kappa+\mu)}\right]. \tag{16}$$

As $\operatorname{Re}\lambda_- < \operatorname{Re}\lambda_+$, fluctuations are damped whenever $\operatorname{Re}\lambda_+ < 0$. When $\operatorname{Re}\lambda_+ > 0$ fluctuations are amplified (grow exponentially) and the base solution is unstable. Hence we are interested in determining whether and when $\operatorname{Re}\lambda_+ = 0$, corresponding to a so-called bifurcation. It is easy to show that for $\Delta/\kappa < -\sqrt{3}$, $\lambda_+ = 0$ (and $\lambda_- = -2\kappa$) at $\mu = \mu^{\uparrow\downarrow}$, with

$$\mu^{\uparrow\downarrow} \equiv \frac{-2\Delta/\kappa \pm \sqrt{(\Delta/\kappa)^2 - 3}}{3}. \tag{17}$$

Note that, whenever Δ/κ is not terribly larger than 1 in modulus, $\mu^{\uparrow\downarrow}$ is of order 1 as well (e.g. for $\Delta/\kappa = -2$, $\mu^\downarrow = 1$ and $\mu^\uparrow = 5/3$). As $K|\alpha_{\text{base}}|^2 = \frac{K\epsilon^2}{\Omega^2}[1+\cos(2\Omega t)] = \kappa\mu[1+\cos(2\Omega t)]$, this proves that $K|\alpha_{\text{base}}|^2 \sim \kappa\mu \sim \kappa$, as needed for the validity of the approximation $\alpha_{\text{base}}(t) \approx -\sqrt{2}(\epsilon/\Omega)\cos(\Omega t)$, which we have used.

It is easy to check that $\lambda_+ > 0$ for $\mu^\downarrow < \mu < \mu^\uparrow$, which implies that the base solution is unstable (fluctuations grow) within that injection region, and is replaced by a new solution. As there λ_+ is real, the emerging solution is characterised by the appearance of a constant contribution on top of $\alpha_{\text{base}}(t)$, which manifests in its spectrum as a component at zero

frequency, physically corresponding to the (uninjected) frequency ω_{L}. Should λ_+ have got an imaginary part (as it happens in the Hopf bifurcation), the emerging solution would be characterised by the emergence of oscillations at that frequency (and some of its harmonics).

Figure S1-a displays the location of the bifurcation boundary (17) as a function of the normalised detuning Δ/κ, together with the bifurcation points numerically found from the complete OM model (2a)-(2c). A good agreement is evident, which supports all previous approximations. Also shown are bifurcation points corresponding to the Hopf bifurcation, which occurs for injections larger than $\mu^{\uparrow}$, as anticipated, and then that bifurcation plays no role in our analysis. Figure S1-b shows an example of the power spectrum below the Hopf bifurcation threshold. Figure S1-c shows a similar spectrum but just above the bifurcation threshold. The red line denotes the location of the carrier frequency ω_L and the two main peaks located at $\omega_L \pm \Omega$ correspond to the driving. The appearance of different peaks in S1-c, at frequencies related with multiples of the mechanical frequency (ω_m), is due to the fact that the fluctuations contain frequency components of the order of ω_m, and there is a mixing with different harmonics of the fundamental mode at frequency Ω.

C. Quadratures fluctuation dynamics

From equation (15) the dynamics of the field fluctuations derives straightforwardly (for $\mu \leq \mu^{\downarrow}$ or $\mu \geq \mu^{\uparrow}$, in this case far below the Hopf bifurcation). We follow the strategy of projecting equation (15) onto the left eigenvectors of its matrix, which we denote by L. When eigenvalues are real (as is our case) such projection leads to equations for physical quadratures, and then is the straightest way to solve the problem[10–12].

The left eigenvectors of matrix L can be written as $\vec{w}_{\pm} = \left(e^{-i\theta_{\pm}}, e^{i\theta_{\pm}}\right)$, where $\theta_+ = -\theta_-$, and both angles can be determined easily by diagonalising L. Projecting equation (15) onto $\vec{w}_{\pm}$ from the left yields decoupled equations for the intracavity quadrature fluctuations $\delta q_{\pm} \equiv \left(e^{-i\theta_{\pm}}\delta a + e^{i\theta_{\pm}}\delta a^{\dagger}\right)$,

$$\delta \dot{q}_{\pm} = \lambda_{\pm}\delta q_{\pm} + \sqrt{2\kappa}q_{\pm,\mathrm{in}}\left(t\right) + \zeta_{\pm}\left(t\right), \tag{18}$$

where $q_{\pm,\mathrm{in}}\left(t\right) = e^{-i\theta_{\pm}}a_{\mathrm{in}}\left(t\right) + e^{i\theta_{\pm}}a_{\mathrm{in}}^{\dagger}\left(t\right)$ is the corresponding quadrature vacuum noise, and

$$\zeta_{\pm}\left(t\right) = -\frac{2\sqrt{2}g_0\epsilon}{\Omega}\sin\left(\theta_{\pm}\right)\cos\left(\Omega t\right)x_T\left(t\right), \tag{19}$$

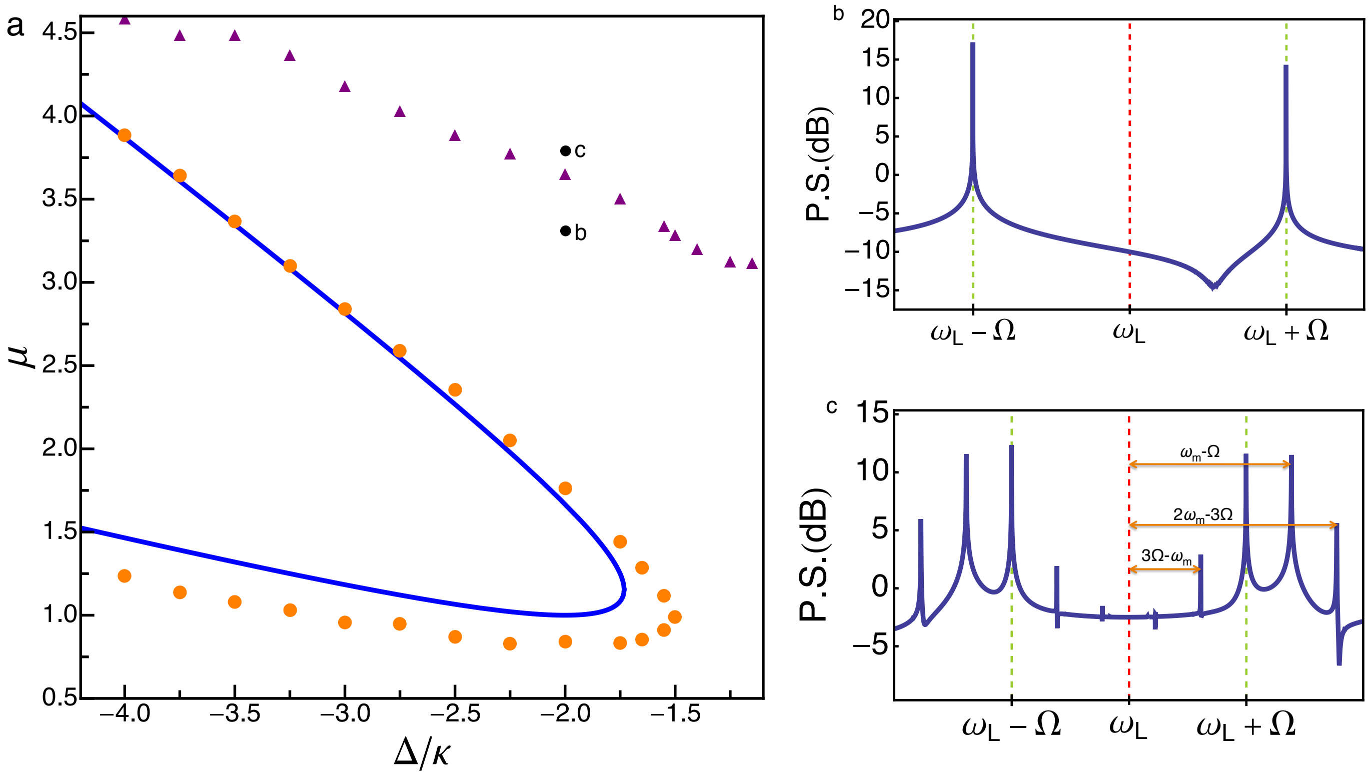

Figure S1 | Instability region. a) The base solution becomes unstable inside the tongue. Spheres denote boundaries of the pitchfork bifurcation obtained from numerical integration of the mean field equations for the complete OM model. Triangles denote the boundary of the Hopf bifurcation. Parameters are $\omega_{\rm m}/\kappa = 30$, $\gamma_{\rm m}/\kappa = 6 \cdot 10^{-6}$ and $\Omega/\kappa = 4\pi$. b) Optical power spectrum (logarithmic sacale) below the Hopf bifurcation threshold for $\Delta/\kappa = -2$ and $\mu = 3.65$. c) Optical power spectrum just above the Hopf bifurcation threshold.

is the mechanical noise coupled to that quadrature. Note that either $\theta_\pm$ should be zero (amplitude quadrature) mechanical noise would have no effect on that quadrature, as is well known for radiation pressure driven optomechanics. The virtue of equation (18) is twofold[10–12]: on one hand we observe that the quadratures $q_\pm$ are the the normal modes of the fluctuations linearised dynamics, and hence form the natural basis for studying field fluctuations; on the other hand, as $-2\kappa \leq \lambda_- < \lambda_+ \leq 0$ close to the bifurcation, q_- (q_+) can be unequivocally indentified with the squeezed (anti-squeezed) quadrature. In particular, at the bifurcation $\lambda_- = -2\kappa$ (its minimum value) and q_- is maximally damped, hence there we expect maximum noise reduction in q_-, while $\lambda_+ = 0$, hence q_+ is undamped and noise

grows without bound (a divergence is expected in its fluctuation).

Equation (18) can be solved straightforwardly, yielding

$$\delta q_{\pm}(t) = \delta q_{\pm}^{\mathrm{vac}}(t) + \delta q_{\pm}^{\mathrm{mec}}(t), \tag{20}$$

$$\delta q_{\pm}^{\mathrm{vac}}(t) = \sqrt{2\kappa}\int_{-\infty}^{t} e^{\lambda_{\pm}(t-t')} q_{\pm,\mathrm{in}}(t')\, dt', \quad \delta q_{\pm}^{\mathrm{mec}}(t) = \int_{-\infty}^{t} e^{\lambda_{\pm}(t-t')}\zeta_{\pm}(t')\, dt', \tag{21}$$

in terms of the noises, whose only non-null two-time correlations read

$$\left\langle q_{\pm,\mathrm{in}}(t)\, q_{\pm,\mathrm{in}}(t')\right\rangle = \delta(t-t'), \tag{22}$$

$$\langle \zeta_{\pm}(t)\,\zeta_{\pm}(t')\rangle = 4\kappa\omega_{\mathrm{m}}\mu \sin^2(\theta_-)\cos(\Omega t)\cos(\Omega t')\langle x_T(t)\, x_T(t')\rangle, \tag{23}$$

where in the last equality we used (14).

D. Squeezing spectrum for the optimum quadrature

The relevant field fluctuations are those carried by the light leaving the cavity. The calculation of outgoing fields follows from the input-output relation of optical cavities[13–17], $a_{\mathrm{out}}(t) + a_{\mathrm{in}}(t) = \sqrt{2\kappa}a(t)$, which exactly transfers to the quadrature fluctuations by linearity:

$$\delta q_{\mathrm{out},\pm}(t) = -q_{\mathrm{in},\pm}(t) + \sqrt{2\kappa}\delta q_{\pm}(t), \tag{24}$$

(remind that $\delta q_{\mathrm{in},\pm}(t) = q_{\mathrm{in},\pm}(t)$ as $\langle q_{\mathrm{in},\pm}(t)\rangle = 0$). The outgoing field is instrinsically multimode (it contains all the modes of the universe), having a continuous spectrum. Hence the appropriate quantity characterising the fluctuations is the spectral variance of the detected quadrature, so-called squeezing spectrum, which is calculated from the two-time correlations

$$C_{\pm}(t,t') \equiv \langle \delta q_{\mathrm{out},\pm}(t)\, \delta q_{\mathrm{out},\pm}(t')\rangle. \tag{25}$$

When $C(t,t')$ is stationary, i.e. when $C(t,t') = C(t-t')$, the squeezing spectrum is just a Fourier transform (Wiener-Kintchine theorem)[15–17]

$$S(\omega) \equiv \int_{-\infty}^{+\infty} C(\tau)\, e^{-i\omega\tau} d\tau, \tag{26}$$

which is normalised so that $S = 1$ corresponds to the standard quantum limit. Squeezing is present when $S < 1$, and (ideally) perfect squeezing, i.e. complete absence of fluctuation, occurs when $S = 0$. When the correlation is not stationary the correct definition is[18]

$$S(\omega) = \frac{1}{T}\int_{-T/2}^{T/2} dt \int_{-T/2}^{T/2} dt' C(t,t')\cos[\omega(t-t')], \tag{27}$$

where T is the measurement time (not to be confused with the temperature). Expression (27) reduces to (26) when the problem is stationary and $T \to \infty$. In our case the mechanical noise correlator is not stationary because of the product $\cos(\Omega t)\cos(\Omega t')$ in (23).

The correlations $C_\pm(t,t')$ are calculated after straightforward algebra as

$$\begin{aligned} C_\pm(t,t') &= \langle q_{\mathrm{in},\pm}(t)\, q_{\mathrm{in},\pm}(t')\rangle - \sqrt{2\kappa}\left[\left\langle q_{\mathrm{in},\pm}(t)\, \delta q_\pm^{\mathrm{vac}}(t')\right\rangle + \left\langle \delta q_\pm^{\mathrm{vac}}(t)\, q_{\mathrm{in},\pm}(t')\right\rangle\right] \\ &+ 2\kappa\left\langle \delta q_\pm^{\mathrm{vac}}(t)\, \delta q_\pm^{\mathrm{vac}}(t')\right\rangle + 2\kappa\left\langle \delta q_\pm^{\mathrm{mec}}(t)\, \delta q_\pm^{\mathrm{mec}}(t')\right\rangle, \end{aligned} \tag{28}$$

where we used that vacuum and mechanical noises are independent (uncorrelated). Each of the pieces can be determined from equations (21), (22), and (23), with the result

$$C_\pm(t,t') = \delta(t-t') - 2\kappa\left(1+\kappa/\lambda_\pm\right) e^{\lambda_\pm |t-t'|} + 8\kappa^2 \omega_{\mathrm{m}} \mu \sin^2(\theta_\pm)\, I_\pm(t,t'), \tag{29}$$

where

$$I_\pm(t,t') = \int_{-\infty}^{t} dt_1 e^{\lambda_\pm (t-t_1)} \cos(\Omega t_1) \int_{-\infty}^{t'} dt_1' e^{\lambda_\pm \left(t'-t_1'\right)} \cos(\Omega t_1') \left\langle x_T(t_1)\, x_T(t_1')\right\rangle, \tag{30}$$

Below we explain how to compute this integral.

Use of definition (27) for large T yields

$$S_\pm(\omega) = 1 + \frac{4\kappa(\kappa+\lambda_\pm)}{\lambda_\pm^2+\omega^2} + 8\kappa^2\omega_{\mathrm{m}}\mu \sin^2(\theta_\pm)\, \mathcal{I}_\pm(\omega), \tag{31}$$

with $\mathcal{I}_\pm(\omega)$ given in (41) below. The above expressions ($\pm$) are obtained assuming $T \gg -1/\lambda_+$, or $T \gg -1/\lambda_-$, which quantifies how large the detection time must be in order for the spectrum $S_+(\omega)$ or $S_-(\omega)$, respectively, to be given by (31).

Finally the squeezing spectrum reads

$$S_\pm(\omega) = 1 + \frac{4\kappa^2\left(1+\lambda_\pm/\kappa\right)}{\lambda_\pm^2+\omega^2} + 2\mu\sin^2(\theta_\pm)\,\frac{1+2n_T}{Q_{\mathrm{m}}}\,\frac{4\kappa^2 \mathrm{X}(\omega;\Omega)}{\lambda_\pm^2+\omega^2}, \tag{32}$$

$$\mathrm{X}(\omega;\Omega) = \frac{1}{2}\left[\left|\chi_{\mathrm{m}}(\omega+\Omega)\right|^2 + \left|\chi_{\mathrm{m}}(\omega-\Omega)\right|^2\right], \tag{33}$$

where $Q_{\mathrm{m}} \equiv \omega_{\mathrm{m}}/\gamma_{\mathrm{m}}$ is the mechanical resonance quality factor, a huge number. Note that for $\omega, \Omega \ll \omega_{\mathrm{m}}$, $\left|\chi_{\mathrm{m}}(\omega\pm\Omega)\right|^2 \approx 1$ and hence $\mathrm{X}(\omega;\Omega) \approx 1$, as used in the main text.

E. Computation of the integral $\mathcal{I}_\pm(\omega)$

Here we explain the computation of the Fourier cosine transform-like integral $\mathcal{I}_\pm(\omega)$ that appears in the expression of the squeezing level, see (31) above, for the non-stationary

correlator case. We refer to a generic eigenvalue λ, and our goal is to compute

$$\mathcal{I}(\omega) \equiv \frac{1}{T} \int_{-T/2}^{T/2} dt \int_{-T/2}^{T/2} dt' I(t,t') \cos\left[\omega(t-t')\right], \tag{34}$$

with

$$I(t,t') = \int_{-\infty}^{t} dt_1 e^{\lambda(t-t_1)} \cos(\Omega t_1) \int_{-\infty}^{t'} dt_1' e^{\lambda\left(t'-t_1'\right)} \cos(\Omega t_1') \langle x_T(t_1) x_T(t_1')\rangle, \tag{35}$$

where λ is an arbitrary complex number. Using the correlation (8) we get,

$$\mathcal{I}(\omega) = \frac{\gamma_{\mathrm{m}}}{\pi\omega_{\mathrm{m}}^2}(1+2n_T) \int_{-\infty}^{+\infty} d\omega_1 \left|\chi_{\mathrm{m}}(\omega_1)\right|^2 G(\omega_1,\omega), \tag{36}$$

where

$$G(\omega_1,\omega) = \frac{1}{T} \iint_{-T/2}^{T/2} dt dt' \cos\left[\omega(t-t')\right] g(\omega_1,t)\, g(-\omega_1,t'), \tag{37}$$

$$g(\omega_1,t) = \int_{-\infty}^{t} e^{\lambda(t-t_1)} \cos(\Omega t_1)\, e^{i\omega_1 t_1} dt_1. \tag{38}$$

The kernel $G(\omega_1,\omega)$ can be caculated analytically. Analysis of its dependence on the frequency ω_1 shows that, in the limit of large T, which is the relevant one as disussed below,

$$G(\omega_1,\omega) \approx \frac{1}{8(\lambda^2+\omega^2)} \sum_{j=1}^{4} T \,\mathrm{sinc}^2\left[\frac{T}{2}(\omega_1-\omega_{\mathrm{res},j})\right], \tag{39}$$

with $\{\omega_{\mathrm{res},j}\}_{j=1}^{4} = \{\Omega+\omega, \Omega-\omega, -\Omega+\omega, -\Omega-\omega\}$, and $\mathrm{sinc}(x) = \sin(x)/x$. The sinc^2 functions multiplied by T act as Dirac deltas for $T\to\infty$,

$$T\,\mathrm{sinc}^2\left[\frac{T}{2}(\omega_1-\omega_{\mathrm{res},j})\right] \approx 2\pi\delta(\omega_1-\omega_{\mathrm{res},j}). \tag{40}$$

Substituting this approximation into equation (36), we get

$$\mathcal{I}(\omega) = \frac{\gamma_{\mathrm{m}}}{\omega_{\mathrm{m}}^2} \frac{1+2n_T}{(\lambda^2+\omega^2)} \frac{\left|\chi_{\mathrm{m}}(\omega+\Omega)\right|^2 + \left|\chi_{\mathrm{m}}(\omega-\Omega)\right|^2}{2}, \tag{41}$$

where we used $\left|\chi_{\mathrm{m}}(-\omega_1)\right|^2 = \left|\chi_{\mathrm{m}}(\omega_1)\right|^2$, see equation (6).

The replacement (40) in equation (36) is correct as far as $\left|\chi_{\mathrm{m}}(\omega_1)\right|^2$ is sufficiently smooth withing an interval of witdth $4\pi/T$ around $\omega_{\mathrm{res},j}$, i.e. when

$$T \gg 4\pi \left|\frac{d}{d\omega_1}\left|\chi_{\mathrm{m}}(\omega_1)\right|^2\right|_{\omega_1=\omega\pm\Omega}. \tag{42}$$

This sets a limit to the time T in order for (41) to be valid.

F. Squeezing spectrum for an arbitrary quadrature θ

In the previous section we have explained how to compute the spectrum of squeezing for the quadratures $\theta_\pm$, being θ_- the optimum squeezed one. This method is valid whenever the quadratures $\theta_\pm$ represent physical quadratures, this fails in our model far from the bifurcation. Here we give the details about the calculation of the spectrum for an arbitrary quadrature $\delta q_\theta \equiv \left(e^{-i\theta}\delta a + e^{i\theta}\delta a^\dagger\right)$, in the direction of $\vec{w}_\theta = (e^{-i\theta}, e^{i\theta})$. We note that the vector for the θ quadrature can be written as a linear combination of the left eigenvectors $\vec{w}_\pm$ ($\mathcal{L}^\top \vec{w}_\pm = \lambda_\pm \vec{w}_\pm$), as $\vec{w}_\theta = B_+\vec{w}_+ + B_-\vec{w}_-$. The coefficients $B_\pm$ can be calculated by projecting with the normalised eigenvectors $\vec{v}_\pm$ (that verify $\vec{w}_\pm \cdot \vec{v}_\pm = 1$ and $\vec{w}_\pm \cdot \vec{v}_\mp = 0$) as $B_\pm = \vec{w}_\theta \cdot \vec{v}_\pm$. The vector of the fluctuations $\delta\vec{A} = \left(\delta a, \delta a^\dagger\right)^\top$ can be written as $\delta\vec{A} = c_+(t)\vec{v}_+ + c_-(t)\vec{v}_-$, where the coefficients $c_\pm$ are

$$c_\pm(t) = \vec{w}_\pm \cdot \delta\vec{A}(t) = \int_{-\infty}^{t} dt_1 e^{\lambda_\pm(t-t_1)} \vec{w}_\pm \cdot \vec{\eta}(t) \tag{43}$$

with $\vec{\eta}(t) = \left(\xi(t), \xi(t)^\dagger\right)^\top$ the vector containing the noises. Thus the expression for the general quadrature is $\delta q_\theta(t) = \vec{w}_\theta \cdot \delta\vec{A}(t) = B_+c_+(t) + B_-c_-(t)$.

Proceeding as in the case for the quadratures $\theta_\pm$, the funcion $C_\theta(t,t') = \langle \delta q_{\text{out},\theta}(t)\delta q_{\text{out},\theta}(t')\rangle$ and the spectrum of squeezing can be computed. Its different terms, in correspondence with equation (28) but for the quadrature θ, are:

$$S_{in-in}(\omega) = 1. \tag{44a}$$

$$S_{in-vac}(\omega) = 2\kappa e^{-i\theta}\left(\frac{B_+w_+^{(2)}}{\lambda_+ + i\omega} + \frac{B_-w_-^{(2)}}{\lambda_- + i\omega}\right). \tag{44b}$$

$$S_{vac-in}(\omega) = 2\kappa e^{i\theta}\left(\frac{B_+w_+^{(1)}}{\lambda_+ - i\omega} + \frac{B_-w_-^{(1)}}{\lambda_- - i\omega}\right). \tag{44c}$$

$$S_{vac-vac}(\omega) = 4\kappa^2\left[\frac{B_+^2 w_+^{(1)}w_+^{(2)}}{\lambda_+^2+\omega^2} + \frac{B_-^2 w_-^{(1)}w_-^{(2)}}{\lambda_-^2+\omega^2} + B_+B_-\left(\frac{w_+^{(1)}w_-^{(2)}}{(\lambda_+ - i\omega)(\lambda_- + i\omega)} + \frac{w_-^{(1)}w_+^{(2)}}{(\lambda_- - i\omega)(\lambda_+ + i\omega)}\right)\right]. \tag{44d}$$

$$S_{mech}(\omega) = -2\mu\kappa^2\frac{(1+2n_T)}{Q_\text{m}}\left[\frac{B_+^2\left(w_+^{(1)} - w_+^{(2)}\right)^2}{\lambda_+^2+\omega^2} + \frac{B_-^2\left(w_-^{(1)} - w_-^{(2)}\right)^2}{\lambda_-^2+\omega^2} + \right.$$
$$\left. + B_+B_-\left(w_+^{(1)} - w_+^{(2)}\right)\left(w_-^{(1)} - w_-^{(2)}\right)\frac{2(\lambda_+\lambda_- + \omega^2)}{(\lambda_+^2+\omega^2)(\lambda_-^2+\omega^2)}\right]. \tag{44e}$$

The total spectrum is, thus: $S_\theta(\omega) = S_{in-in} + S_{in-vac} + S_{vac-in} + S_{vac-vac} + S_{mech}$, and the symbols $w_\pm^{(i)}$ with $i = 1, 2$ represent the i component of the vector $\vec{w}_\pm$.

In order to obtain the last result we had to compute the following terms $\langle c_{\pm}^{\text{mech}}(t)c_{\mp}^{\text{mech}}(t')\rangle$, that needed a generalization of the integral (35) for the case with different eigenvalues in the exponentials. The definition of $\mathcal{I}_{\pm}(\omega)$, is the same as in equation (34) but substituting the $G(\omega_1,\omega)$ function by the new one $G_{\pm}(\omega_1,\omega)$, given by

$$G_{\pm}(\omega_1,\omega)=\frac{1}{T}\iint_{-T/2}^{T/2}dtdt'\cos\left[\omega\left(t-t'\right)\right]\left\{g_{+}\left(\omega_1,t\right)g_{-}\left(-\omega_1,t'\right)+g_{-}\left(\omega_1,t\right)g_{+}\left(-\omega_1,t'\right)\right\}, \tag{45}$$

$$g_{\mathrm{i}}\left(\omega_1,t\right)=\int_{-\infty}^{t}e^{\lambda_{\mathrm{i}}(t-t_1)}\cos\left(\Omega t_1\right)e^{i\omega_1 t_1}dt_1. \tag{46}$$

The kernel $G_{\pm}(\omega_1,\omega)$ can be calculated analytically, in the limit of large T

$$G_{\pm}\left(\omega_1,\omega\right)\approx\frac{\lambda_{+}\lambda_{-}+\omega^2}{4\left(\lambda_{+}^2+\omega^2\right)\left(\lambda_{-}^2+\omega^2\right)}\sum_{k=1}^{4}T\,\mathrm{sinc}^2\left[\frac{T}{2}\left(\omega_1-\omega_{\mathrm{res},k}\right)\right], \tag{47}$$

with $\{\omega_{\mathrm{res},k}\}_{k=1}^{4}=\{\Omega+\omega,\Omega-\omega,-\Omega+\omega,-\Omega-\omega\}$, and $\mathrm{sinc}\left(x\right)=\sin\left(x\right)/x$. Using the approximation for $T\to\infty$ we have

$$\mathcal{I}_{\pm}(\omega)=\frac{2\left(1+2n_T\right)\gamma_{\mathrm{m}}}{\omega_{\mathrm{m}}^2}\frac{\lambda_{+}\lambda_{-}+\omega^2}{\left(\lambda_{+}^2+\omega^2\right)\left(\lambda_{-}^2+\omega^2\right)}\mathrm{X}(\omega;\Omega). \tag{48}$$

As an example we plot the minimum value of the spectrum of squeezing in function of the injection parameter μ in the region $\mu<\mu^{\downarrow}$ and $\mu>\mu^{\uparrow}$, for each μ the quadrature that is maximally squeezed changes. As it can be seen from Figure S2-a,b the minimum squeezing attainable decreses as the system is moved from the lower bifurcation $\mu^{\downarrow}$ (S2-a) or from the upper bifurcation $\mu^{\uparrow}$ (S2-b).

III. THE COMPLETE OM MODEL NUMERICAL ANALYSIS

In order to verify our analytical results we have simulated the complete OM model (2) in order to determine both its mean field solutions and the quantum fluctuations affecting them.

A. Mean field solutions

Taking expectation values in model (2) and using the standard semiclassical approximation (neglection of quantum fluctuations around the mean values), the OM model can be

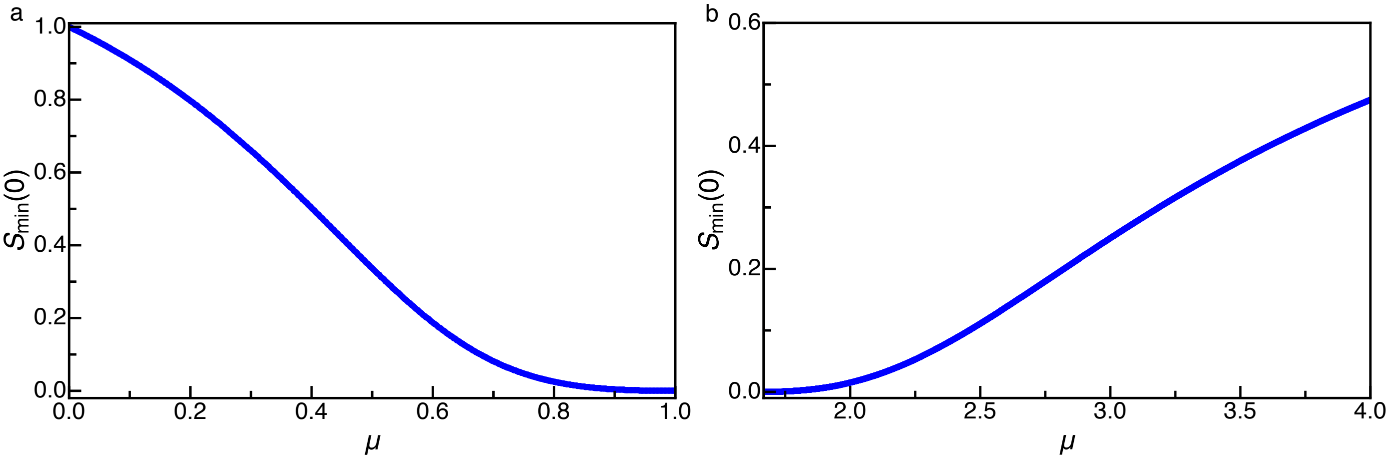

Figure S2 |Minimum of the spectrum of squeezing. The minimum of the spectrum of squeezing at $\omega = 0$ is plotted as a function of the injection parameter μ for the same parameters as in Fig. S1. a) Below $\mu^{\downarrow}$ and b) above $\mu^{\uparrow}$.

written in terms of scaled time and variables,

$$\tau = \kappa t, \quad X = \frac{g_0}{\kappa} \langle x \rangle, \quad P = \frac{g_0}{\kappa} \langle p \rangle, \quad A = \frac{g_0}{\kappa} \langle a \rangle, \tag{49}$$

and using the dimensionless injection parameter μ defined in (14), as the following set of dimensionless equations:

$$dX/d\tau = \bar{\omega}_{\rm m} P, \tag{50a}$$

$$dP/d\tau = -\bar{\gamma}_{\rm m} P - \bar{\omega}_{\rm m} X + 2 |A|^2, \tag{50b}$$

$$dA/d\tau = -A + i \left(\bar{\Delta} + X \right) A + \sqrt{\mu \bar{\omega}_{\rm m}} \bar{\Omega} \sin \left(\bar{\Omega} \tau \right), \tag{50c}$$

which contain 5 dimensionless parameters:

$$\bar{\omega}_{\rm m} = \frac{\omega_{\rm m}}{\kappa}, \; \bar{\gamma}_{\rm m} = \frac{\gamma_{\rm m}}{\kappa}, \; \bar{\Omega} = \frac{\Omega}{\kappa}, \; \bar{\Delta} = \frac{\Delta}{\kappa}, \; \mu = \frac{2 g_0^2 \epsilon^2}{\kappa \omega_{\rm m} \Omega^2}. \tag{51}$$

(50) is the system of equations that we use to perform the numerical simulations. Note in particular that the value of the OM coupling constant g_0 does not appear in the equations, only through the combined injection parameter μ. Of course, different values of g_0 will correspond to different values of the injection power ϵ^2 in order to yield a given value of μ; but that is all its influence.

In Figure S3 we plot the results of the numerical simulation of equation (50) for a given set of parameters. From Figure S3-a it can be seen that the mirror suffers a mean shift

from its equilibrium position. The quality of our analytical approximation obtaining the intracavity mean field α_{base} from equation (11) can be checked from Figure S3-b, where the numerical result of the complete optomechanical model $|A(\tau)|^2$ is compared with the analytical one. In panel S3-c the change of behaviour in the intracavity intensity just above the bifurcation is observed.

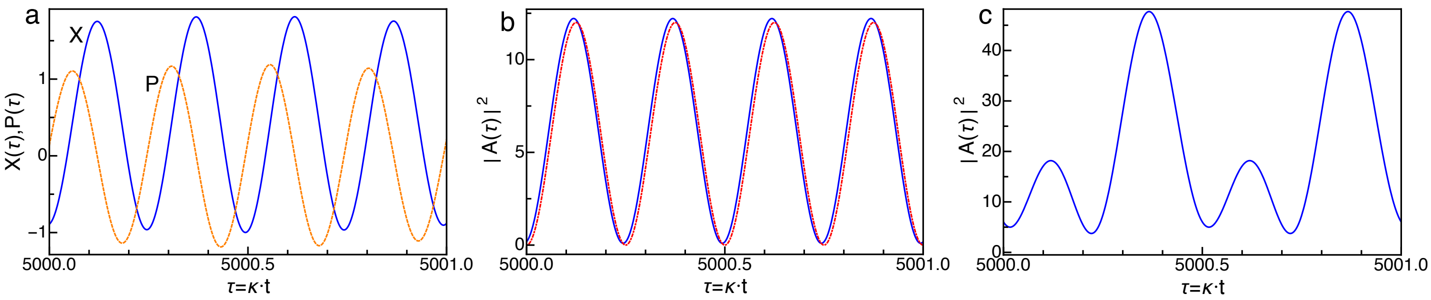

Figure S3 |Mean field solutions. Numerical results of the following scaled variables given in equation (50): a) $X(\tau)$ (blue) and $P(\tau)$ (dashed orange) for $\mu = 0.4$. b) $|A(\tau)|^2$ far from the bifurcation and tha analytical prediction $\alpha^2_{\mathrm{base}}(\tau)$ (red dashed) at $\mu = 0.4$. c) $|A(\tau)|^2$ near the bifurcation threshold at $\mu = 0.9$. Parameters are identical to those of Fig. S1.

B. Quantum fluctuations

In order to study the dynamics of quantum fluctuations around the mean value solutions ($x \to \langle x\rangle + \delta x$, etc.) we use the standard linear approximation, consisting in neglecting terms quadratic or of higher order in the fluctuations. Note that this approximation is reasonable if the mean values are large as compared with the fluctuations. The mean values read, see (49),

$$\langle x\rangle = \frac{\kappa}{g_0}X, \quad \langle p\rangle = \frac{\kappa}{g_0}P, \quad \langle a\rangle = \frac{\kappa}{g_0}A, \tag{52}$$

in terms of the normalised variables X, P, and A, which are of order 1 as we have seen in the previous discussion. Thus, whenever the ratio $\frac{\kappa}{g_0} \gg 1$, as usual[2,3], the mean values are very large as compared with the fluctuations, which are of order 1 because noise terms are too.

We normalise time as $\tau = \kappa t$ as before, obtaining the following system of equations, which

we write in matrix form for convenience:

$$\frac{d}{d\tau}\mathbf{D} = \mathcal{L}_{\mathrm{OM}}(\tau)\cdot\mathbf{D} + \mathbf{N}(\tau), \tag{53}$$

where

$$\mathbf{D}(\tau) = \begin{pmatrix} \delta x(t) \\ \delta p(t) \\ \delta a(t) \\ \delta a^{\dagger}(t) \end{pmatrix}, \tag{54}$$

$$\mathcal{L}_{\mathrm{OM}}(\tau) = \begin{pmatrix} 0 & \bar{\omega}_{\mathrm{m}} & 0 & 0 \\ -\bar{\omega}_{\mathrm{m}} & -\bar{\gamma}_{\mathrm{m}} & 2A^{*}(\tau) & 2A(\tau) \\ iA(\tau) & 0 & -1+i\left[\bar{\Delta}+X(\tau)\right] & 0 \\ -iA^{*}(\tau) & 0 & 0 & -1-i\left[\bar{\Delta}+X(\tau)\right] \end{pmatrix}, \tag{55}$$

and

$$\mathbf{N}(\tau) = \begin{pmatrix} 0 \\ \sqrt{2\bar{\gamma}_{\mathrm{m}}}\bar{\eta}(t) \\ \sqrt{2}\bar{a}_{\mathrm{in}}(\tau) \\ \sqrt{2}\bar{a}_{\mathrm{in}}^{\dagger}(\tau) \end{pmatrix}, \tag{56}$$

where

$$\bar{\eta}(\tau) = \eta(t)/\sqrt{\kappa}, \quad \bar{a}_{\mathrm{in}}(\tau) = a_{\mathrm{in}}(t)/\sqrt{\kappa}, \quad \bar{a}_{\mathrm{in}}^{\dagger}(\tau) = a_{\mathrm{in}}^{\dagger}(t)/\sqrt{\kappa}, \tag{57}$$

are scaled quantum noises. The information we need about them is in their correlators, which read exactly as the original ones, see (3), with $t, t' \to \tau, \tau'$. Note again that g_0 does not appear in the quantum fluctuation equations.

In order to find numericaly the solutions to Eq. (53), since the system is not stationary due to the time dependence of the base solution, we proceed as follows. First we compute the desired mean field solution from (50), which is substituted into matrix $\mathcal{L}_{\mathrm{OM}}(\tau)$. Then, as Eq. (53) is linear, it can be solved formally as explained in, e.g.[19]. Finally, from the formal solution, any two-time correlation can be computed numerically by using the noise correlators (3).

---

[1] Kippenberg, T. J. & Vahala, K. J. Cavity opto-mechanics. *Opt. Express* **15,** 17172- 17205 (2007).

[2] Milburn, G. J. & Woolley, M. J. An introduction to quantum optomechanics. *Acta Phys. Slovaca* **61,** 483–601 (2011).

[3] Aspelmeyer, M. Kippenberg, T. J. & Marquardt, F. Cavity optomechanics. *Rev.Mod.Phys.* **86,** 1391-1452 (2014).

[4] Fabre, C. *et al.* Quantum-noise reduction using a cavity with a movable mirror. *Phys. Rev. A* **49,** 1337-1343 (1994).

[5] Mancini, S. & Tombesi, P. Quantum noise reduction by radiation pressure. *Phys. Rev. A* **49,** 4055-4065 (1994).

[6] Marquardt, F. & Girvin, S. M. Optomechanics. *Physics* **2,** 40 (2009).

[7] Kippenberg, T. J. Rokhsari, H. Carmon, T. Scherer, A. & Vahala, K. J. Analysis of radiation-pressure-induced mechanical oscillation of an optical microcavity. *Phys. Rev. Lett.* **95,** 033901 (2005).

[8] Metzger, C. *et al.* Self-induced oscillations in an optomechanical system. *Phys. Rev. Lett.* **101,** 133903 (2008).

[9] Ludwig, M. Kubala, B. & Marquardt, F. The optomechanical instability in the quantum regime. *New. J. Phys.* **10,** 095013 (2008).

[10] Pérez-Arjona, I. Roldán, E. & de Valcárcel, G. J. Quantum squeezing of optical dissipative structures. *Europhys. Lett.* **74,** 247-253 (2006).

[11] Pérez-Arjona, I. Roldán, E. & de Valcárcel, G. J. Theory of quantum fluctuations of optical dissipative structures and its application to the squeezing properties of bright cavity solitons. *Phys. Rev. A* **75,** 063802 (2007).

[12] Navarrete-Benlloch, C. Roldán, E. & de Valcárcel, G. J. Noncritically squeezed light via spontaneous rotational symmetry breaking. *Phys. Rev. Lett.* **100,** 203601 (2008).

[13] Collett, M. J. & Gardiner, C. W. Squeezing of intracavity and traveling-wave light fields produced in parametric amplification. Phys. Rev. A **30**, 1386-1391 (1984).

[14] Gardiner, C. W. & Collett, M. J. Input and output in damped quantum systems: Quantum stochastic differential equations and the master equation. Phys. Rev. A **31**, 3761-3774 (1985).

[15] Walls, D. F. & Milburn, G. J. *Quantum Optics* (Springer, Berlin, 2008).

[16] Gardiner, C. W. & Zoller, P. *Quantum Noise* (Springer, Berlin, 2004).

[17] Carmichael, H. J. *Statistical Methods in Quantum Optics 2* (Springer, Berlin, 2008).

[18] Gea-Banacloche, J. *et al.* Treatment of the spectrum of squeezing based on the modes of the

universe. I. Theory and a physical picture. *Phys. Rev. A* **41,** 369-380 (1990).

[19] Mari, A. & Eisert, J. Gently modulating optomechanical systems. *Phys. Rev. Lett.* **103**, 213603 (2009).
